# A Comparison of Comprehensive HIV/AIDS Knowledge Among Women Across Seven Post-Soviet Countries

**DOI:** 10.5195/cajgh.2018.295

**Published:** 2018-01-30

**Authors:** Hakim Zainiddinov, Nazim Habibov

**Affiliations:** 1 Rutgers University, United States; 2 University of Windsor, Canada

**Keywords:** HIV infection, comprehensive HIV/AIDS knowledge, women, heterosexual transmission, post-Soviet countries

## Abstract

**Introduction:**

Post-Soviet countries of Eastern Europe and Central Asia have witnessed a recent growth of HIV infection through heterosexual transmission. Women’s low levels of knowledge about HIV prevention and transmission methods have been found to account for the higher female-to-male ratio among cases infected through the heterosexual route. This cross national comparison study assessed comprehensive HIV/AIDS knowledge and its key determinants among women of seven post-Soviet countries and identified which countries face the highest levels of risk due to the low levels of HIV/AIDS awareness.

**Methods:**

Study data were obtained from the third wave of the Multiple Indicator Cluster Surveys (MICS3) (conducted in 2005 and 2006), nationally representative samples of women aged 15–49 years. Data on HIV/AIDS knowledge were analyzed for women in Kazakhstan (N=14,310), Kyrgyzstan (N=6,493), Tajikistan (N=4,676), Uzbekistan (N=13,376), Belarus (N=5,884), Ukraine (N=6,066), and Georgia (N=7,727) using descriptive statistics and ordinary least squares (OLS) regressions.

**Results:**

We found that the percentage of women who could correctly identify all five modes of HIV/AIDS transmission and prevention was highest in Eastern European countries of Belarus (34.98%) and Ukraine (31.67%). Across all countries, the strongest predictors of comprehensive HIV/AIDS knowledge were age, education, and region of residence. Marital status, area of residence (urban vs. rural), and household wealth were significant predictors for several countries.

**Conclusion:**

High rates of comprehensive HIV/AIDS knowledge were found among women of Belarus and Ukraine. To reduce the spread of HIV in the region, programs promoting comprehensive HIV/AIDS knowledge for women of younger ages and with lower education are recommended.

In the past fifteen years many countries around the world witnessed dramatic decline in HIV incidence and mortality.^1^ Between 2001 and 2011, the number of people acquiring HIV declined by 50% in 25 low and middle-income countries.^1^ Nepal was at the top of the list with a drop of 91%, followed by Ethiopia at 90%, Cambodia at 88%, Suriname at 86%, Myanmar at 74%, Dominican Republic at 73%, Malawi at 72%, and Botswana at 71%.^1^ Yet, these encouraging national trends in a dramatic reduction in the rate of new HIV infections in many parts of the world were not observed in Eastern Europe and Central Asia.^1^,^2^ For example, Georgia, Kazakhstan, and Kyrgyzstan reported a 25% increase in the rate of new HIV infections between 2001 and 2011.^1^ According to recent WHO report, between 2006 and 2012, rates of HIV diagnoses per 100 000 population increased more than threefold in Tajikistan, twofold in Kyrgyzstan, and by 72% and 92% in Belarus and Georgia, respectively.^3^ Among 52 countries of the WHO European Region, Ukraine reported the highest rate (37.1%) of newly diagnosed HIV infections in 2012.^3^ Several other countries of Eastern Europe and Central Asia, including Belarus (13.1%), Kyrgyzstan (12.8%), and Kazakhstan (12.4%) were among the countries with the highest rates of newly diagnosed HIV infections.^3^ Political and economic instability, as well as the collapse of highly structured public health system contribute to growing rates of HIV/AIDS epidemic throughout Central Asia and Eastern Europe.^2^,^4^ Other factors leading to the high HIV prevalence rates in post-Soviet countries include low levels of preventive practices and punitive measures taken against HIV infected people.^5^ Among low and middle-income countries, the treatment gap remains one of the highest for countries of Eastern Europe and Central Asia. In 2011, there was a 75% gap between the number of people receiving antiretroviral therapy (130,000) and the number of people eligible for treatment (510,000).^1^

In the past decades, the leading mode of HIV transmission in Eastern Europe and Central Asia changed from injection drug route to heterosexual transmission.^2^,^3^ Between 2006 and 2012, the number of HIV infections acquired through heterosexual transmission increased more than three times in Kazakhstan and Kyrgyzstan, and around six times in Tajikistan.^3^ In 2012, the majority (60%) of new HIV infections were acquired through heterosexual contact in 13 of the 15 countries in the Eastern part of the WHO European Region.^3^ Heterosexual mode of transmission affects women more than men.^3^

One of the main factors contributing to the higher female-to-male ratio among cases infected through heterosexual transmission is a low level of knowledge on HIV prevention and transmission methods among women. Recent nationally representative surveys conducted in 26 of 31 countries with generalized epidemics revealed that less than 50% of women have comprehensive HIV knowledge.^1^ Similarly, empirical studies demonstrate that although women’s awareness about HIV/AIDS has increased, their levels of comprehensive knowledge, as defined by UNICEF, either remain low,^6^,^7^ or have not reached the 90% targeted threshold set by the United Nations General Assembly Special Session (UNGASS).^8^

Since injection drug use was the primary method of HIV transmission in the early days of the epidemic, it is not surprising that most published studies of HIV infections in Eastern Europe and Central Asia were focused on injection drug users.^9^,^20^,^11^,^12^ Given the recent rise of new HIV infections acquired through heterosexual contact, this study examined women’s comprehensive HIV knowledge in post-Soviet countries to determine regional differences and identify key determinants that could serve in guiding future policies aimed at reducing discrepancies across countries and regions. We focused this investigation on seven post-Soviet countries, including four Central Asian (Kazakhstan, Kyrgyzstan, Tajikistan, and Uzbekistan), two Eastern European (Belarus and Ukraine), and one Caucasian (Georgia). The selection of these countries was determined by 1) data availability; and 2) representativeness of the former Soviet Union.

The HIV/AIDS trends vary in each of these countries. In 2012 the percentage of newly diagnosed HIV infections ranged from the lowest 10.2% in Tajikistan to the highest 37.1% in Ukraine.^3^ The levels of public health expenditure differ substantially across these countries. In 2006, the levels of public health expenditure were lower in the countries of the Caucasus and Central Asia (less than 3% of GDP and even below 1% in Georgia and Tajikistan) and higher in the Western Commonwealth of Independent Countries (CIS) countries (4% and 5% of GDP), which includes Belarus and Ukraine.^13^

Given the level of socio-economic development of Central Asian countries (except for Kazakhstan, the other three countries are low middle income countries^13^) and their low levels of public health expenditure, it is expected that women of this region will report lower levels of comprehensive HIV knowledge compared to women from Eastern Europe.

## Methods

### Data sources

Study data were obtained from the third wave of the Multiple Indicator Cluster Surveys (MICS3) (conducted in 2005 and 2006), nationally representative samples of women aged 15–49 years. Eligible women were interviewed from the list of households chosen for participation in the surveys.^14^,^15^,^16^,^17^,^18^,^19^ The survey samples were selected using a multi-stage stratified cluster sampling approach.^14^,^15^,^16^,^17^,^18^,^19^ The data were collected in more than 50 countries around the world.^14^,^15^,^16^,^17^,^18^,^19^ MICS3 datasets provide information about basic household socio-demographic characteristics, nutrition, and health indicators for women and children, and women’s general knowledge about HIV/AIDS, as well as its prevention and transmission methods. The data are publicly available and can be accessed at MICS website.^20^ MICS3 surveys included a large number of post-Soviet countries: four Central Asian countries (Kazakhstan, Kyrgyzstan, Tajikistan, and Uzbekistan), two Eastern European countries (Belarus and Ukraine), and one country from the Caucasus region (Georgia). The data for other MICS waves were available for a limited number of post-Soviet countries, thus our study focused on the wave with the most complete data available. The total sample for all seven countries is 58,532, out of which 4,676 come from Tajikistan, 5,884 from Belarus, 6,066 from Ukraine, 6,493 from Kyrgyzstan, 7,727 from Georgia, 13,376 from Uzbekistan, and 14,310 from Kazakhstan. Women’s response rates in the surveys were very high, with 90.3% for Georgia, 96% for Tajikistan, 98% for Uzbekistan, 99% for Kazakhstan and Kyrgyzstan, and 99.8% for Belarus and Ukraine. Statistical Agencies of the analyzed countries conducted MICS3 with the financial and technical support of the United Nations Children’s Fund (UNICEF).

### Measures

Using the UNICEF definition of comprehensive knowledge of HIV/AIDS that includes an accurate identification of two primary methods of HIV prevention (using condoms and having one uninfected partner) and rejection of three common misconceptions about HIV transmission (HIV can be transmitted by sharing food and by mosquito bites, and a healthy-looking person cannot be infected),^14^,^15^,^16^,^17^,^18^,^19^ we created a scale, labeled “comprehensive HIV/AIDS knowledge.” The scale ranged from 0 to 5, with higher scores indicating high comprehensive HIV/AIDS knowledge, and lower scores indicating low comprehensive knowledge about the disease. We estimated Cronbach’s alphas to show the degree of consistency in our scale. Cronbach’s alphas ranging from the lowest to highest were as follows: 0.34 for Belarus, 0.43 for Ukraine, 0.48 for Kyrgyzstan and Kazakhstan, 0.50 for Georgia, 0.52 for Uzbekistan, and 0.56 for Tajikistan. Except for Belarus, alpha coefficients for other countries were within the range of previous studies.^21^,^22^

Independent variables included several sociodemographic measures that were potentially correlated with AIDS knowledge.^23^ Demographic measures include: age (between 15–49), marital status (currently married vs. not married), region of residence (capital city vs. other), and area of residence (rural vs. urban). Education was classified as (higher degree (university graduate) vs. other), and level household income (recoded as tertiles: lower income, middle income, upper income).

### Statistical analysis

Univariate analyses were used to identify associations between the variables across seven post-Soviet countries. To evaluate comprehensive HIV/AIDS knowledge and its key determinants among women, we ran ordinary least squares (OLS) regression on the weighted data. To accurately assess the effect of age, which might not have a linear relationship with the outcome measure, we also included the age squared variable into our models. Data analyses were conducted using Stata 14 statistical software.

## Results

Respondents’ average comprehensive knowledge of HIV ranged from the lowest 2.60 (SD = 1.49) in Tajikistan to the highest 3.86 (SD = 1.08) in Belarus (Table 1). Average age ranged from 29.45 (SD = 9.97) in Uzbekistan to 32.31 (SD = 9.89) in Georgia. The majority of participants, ranging from 57.88% in Kazakhstan to 70.87% in Belarus, were currently married. A small percentage of respondents, ranging from 5.29% in Kazakhstan to 24.21% in Tajikistan, resided in the capital city.

Except for Uzbekistan (41.61%) and Tajikistan (43.69%), the majority of respondents in other countries resided in urban areas. Women in Uzbekistan (10.05%) and Tajikistan (17.39%) comprised the lowest percentage of respondents with higher (university/institute graduate) levels of education, whereas women in Georgia (39.11%) and Ukraine (43.54%) had the highest percentage. The percentage of household wealth was evenly distributed among three categories across all countries, except for Tajikistan and Georgia.

**Figure 1:**
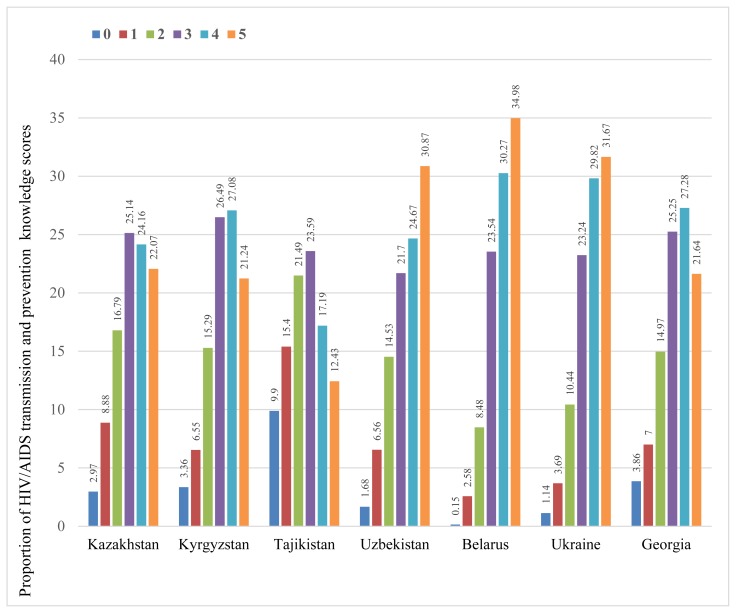
Distribution of scores for HIV/AIDS knowledge across seven compared countries.

The proportion of women who could not correctly identify a single mode of HIV/AIDS transmission and prevention was found to be the highest among women in Tajikistan (9.9%) and lowest among women in Belarus (0.15%) (Figure 1). One third of the respondents in Uzbekistan (30.87%), Ukraine (31.67%), and Belarus (34.98%), every fifth respondent in Kazakhstan (22.07%), Kyrgyzstan (21.24%), and Georgia (21.64%), and only 12.43% of respondents in Tajikistan could correctly identify all five modes of HIV/AIDS transmission and prevention. Similarly across the countries of interest, over 20% of women could correctly identify three modes of transmission and prevention.

Across all of the analyzed countries, younger women’s comprehensive HIV knowledge was higher compared to older women (Table 2). Currently married women in Uzbekistan reported comprehensive HIV knowledge scores that were 0.03 points higher than non-married women (p ≤ 0.01), whereas married women in Kyrgyzstan and Ukraine reported scores that were 0.04 points lower than non-married women (p ≤ 0.05). Region of residence was inversely related to women’s comprehensive HIV knowledge in Uzbekistan, Belarus, Ukraine, and Georgia, whereas women residing in the capital cities of Kazakhstan, Kyrgyzstan, and Tajikistan reported scores that were respectively 0.04, 0.38, and 0.15 points higher than those residing in one of the regions outside of the capital cities. Women from rural areas in Kazakhstan and Georgia reported scores that were respectively 0.03 and 0.07 points lower, and rural women from Kyrgyzstan reported scores that were 0.04 points higher than women from urban areas. Education was positively related with women’s comprehensive HIV knowledge across all countries. Household wealth had a positive significant association with comprehensive HIV knowledge for women only in one Caucasian (Georgia) and three Central Asian (Kazakhstan, Tajikistan, and Uzbekistan) countries (p ≤ 0.001).

## Discussion

As expected, the lowest levels of comprehensive HIV knowledge were found among respondents in Central Asia, whereas the highest levels were reported by women from Eastern European countries. The difference between Belarus with the highest levels and Tajikistan with the lowest levels of comprehensive knowledge was almost threefold. Uzbekistan was an exception among Central Asian countries. The percentage of women in Uzbekistan who could identify all five modes of HIV transmission and prevention was the third highest among the seven analyzed countries. It could be partially explained by the high levels of the country’s public expenditure on education. In 2006, among the 26 analyzed countries of Central and Eastern Europe and CIS, Uzbekistan reported the highest level of expenditure on education (6.3% of GDP).^13^

Overall, the level of comprehensive HIV knowledge among women in all post-Soviet countries under investigation remains low. As a comparison, in 2008–2009, 54% of young urban women in Kenya, a country greatly affected by AIDS pandemic, reported having comprehensive HIV knowledge.^8^ This difference can be attributed to the fact that Sub-Saharan Africa has been heavily affected by HIV/AIDS and consequently attracted substantial global interest and funding for HIV/AIDS treatment, raising the population awareness. Additionally, variations in public spending on HIV/AIDS can account for the difference. In 2001–2005, there was a moderate increase of 30% and 10% in domestic public expenditure on HIV/AIDS from governments of lower-middle-income and upper-middle-income countries respectively, whereas sub-Saharan African countries witnessed an increase of 130% during the same time period.^24^

Among socio-demographic characterstics, we found that age, education, and region were strongly associated with women’s comprehensive HIV knowledge across all countries. With increase in age, women’s comprehensive HIV knowledge also increased. Yet, this positive effect declined with advanced age. One speculation is women’s loss of interest in the subject, due to their decreasing levels of sexual activity. Another explanation can be linked to cohort differences. The older women may be less likely to have received the same information or public health messaging about HIV when compared to the younger women.

Compared to less educated women, women with higher education reported higher levels of comprehensive HIV/AIDS knowledge. This finding is consistent with previous studies^6^,^7^,^8^, and also supported by ethnographic research demonstrating that highly educated women possess more detailed knowledge about HIV/AIDS.^25^

Interestingly, the association between comprehensive HIV knowledge and the capital city region of residence was positive among women in Kazakhstan, Kyrgyzstan, and Tajikistan, and negative among women in Uzbekistan, Georgia, Ukraine, and Belarus. This differential effect could be attributed to varying degrees of access to information and healthcare services. One may speculate that women residing in the capital cities of Kazakhstan, Kyrgyzstan, and Tajikistan have better access to information about HIV/AIDS, are more aware of HIV, and resort to HIV testing compared to respondents from Uzbekistan, Georgia, Ukraine, and Belarus.

The effects of several covariates on comprehensive HIV knowledge varied from country to country. Being currently married increased comprehensive HIV knowledge among women in Uzbekistan, but had a reverse effect on women’s knowledge in Kyrgyzstan and Ukraine. Prior research suggests that married women might benefit from their husbands’ knowledge, as it can be a case for Uzbekistan. However, they can also take fewer precautions in their marital sex, neglecting the possibility of their husbands’ sexual relationships outside of marriage.^8^

Mixed results were also found for the area of residence. Living in rural areas decreased comprehensive HIV knowledge for women in Kazakhstan and Georgia, which is consistent with prior studies.^6^ Yet, for women from Kyrgyzstan the association was reverse. The positive association between rural area and comprehensive HIV knowledge can be linked to high levels of labor migration among rural dwellers. Experts have already raised alarms about the growing numbers of HIV infections among labor migrants, and growing risk for HIV transmission to their home countries.^26^

Household wealth is positively associated with comprehensive HIV knowledge, although the effects were mostly significant for Central Asian countries. Respondents in the middle and upper income tertiles reported significantly higher levels of comprehensive HIV knowledge than those in the lower income tertile. This finding corroborates previous research.^6^ Insignificant effects of wealth on comprehensive HIV knowledge in other countries under investigation are consistent with another group of studies and can be attributed to the association betweeb wealth and education, another proxy of social status.^7^

The present study has several limitations. First, the exploratory nature and scope of the present study do not allow us to include factors beyond sociodemographic characteristics. Future research can explore other potential factors, such as access to HIV testing and care, knowledge of HIV status, and exposure to information on HIV prevention and transmission that could influence women’s comprehensive HIV knowledge. Second, although Cronbach’s alphas for created scales are within the range of previous studies, they are relatively low. Upon availability of other surveys producing scales with relatively high degrees of internal consistency, the study should be replicated to see whether the observed patterns persist across time periods and countries. Future studies should also investigate clinical and public health significance of these findings.

The recent growth of HIV infection through heterosexual transmission in post-Soviet countries of Eastern Europe and Central Asia, especially among women, is alarming. This study revealed differences in the comprehensive HIV knowledge, but it was low among the studied countries. Across all countries, the strongest predictors of comprehensive HIV/AIDS knowledge were age, education, and region of residence. The percentage of women who could correctly identify all five modes of HIV/AIDS transmission and prevention was highest in Belarus (34.98%) and Ukraine (31.67%), suggesting that knowledge increases as the population level of the HIV epidemic increases.

## Figures and Tables

**Table 1:** Descriptive Statistics for the Sample

	Kazakhstan (n=14,310) Mean (SD) or %	Kyrgyzstan (n=6,493) Mean (SD) or %	Tajikistan (n=4,676) Mean (SD) or %	Uzbekistan (n=13,376) Mean (SD) or %	Belarus (n=5,884) Mean (SD) or %	Ukraine (n=6,066) Mean (SD) or %	Georgia (n=7,727) Mean (SD) or %
**Comprehensive**							
**HIV/AIDS knowledge**	3.25 (1.36)	3.31 (1.32)	2.60 (1.49)	3.54 (1.32)	3.86 (1.08)	3.72 (1.18)	3.30 (1.35)
**Age (range: 15–49)**	31.36 (10.32)	29.56 (10.08)	30.47 (9.48)	29.45 (9.97)	31.49 (9.35)	30.65 (9.53)	32.31 (9.89)
**Marital status**							
Currently married	57.88	60.88	65.44	64.27	70.87	68.08	63.50
Not married	42.12	39.12	34.56	35.73	29.13	31.92	36.50
**Region of residence**							
Capital	5.29	14.60	24.21	14.66	16.04	5.87	18.18
Other	94.71	85.40	75.79	85.34	83.96	94.13	81.82
**Area**							
Urban	52.65	58.91	43.69	41.61	68.13	63.57	59.60
Rural	47.35	41.09	56.31	58.39	31.87	36.43	40.40
**Education**							
Higher	25.60	23.78	17.39	10.05	25.19	43.54	39.11
Other	74.40	76.22	82.61	89.95	74.81	56.46	60.89
**Household wealth**							
Lower income	33.11	32.51	25.58	33.63	33.21	33.84	30.93
Middle income	32.82	32.62	30.33	33.27	33.38	33.55	26.08
Upper income	34.07	34.87	44.10	33.10	33.41	32.61	42.99

Notes: Means and standard deviations are presented for continuous variables; proportions (%) are presented for categorical variables. Due to differences in the number of regions across countries, only capital city is shown.

**Table 2:** Sociodemographic characteristics predicting| comprehensive HIV/AIDS knowledge across seven countries.

	Kazakhstan		Kyrgyzstan		Tajikistan		Uzbekistan		Belarus		Ukraine		Georgia	

	B	SE	B	SE	B	SE	B	SE	B	SE	B	SE	B	SE
**Age (range: 15–49)**	0.39	0.009***	0.45	0.02***	0.45	0.02***	0.49	0.01***	0.34	0.01**	0.48	0.01***	0.40	0.01***
**Age square**	−0.37	0.0001***	−0.41	0.0002***	−0.39	0.0003**	−0.45	.0001***	−0.38*	0.0002*	−0.45	0.0002***	−0.37	0.0002***
**Marital status**														
Currently married^a^	0.008	0.03	−0.04	0.05*	−0.02	0.07	0.03	0.04**	0.01	0.04	−0.04	0.04*	−0.004	0.04
**Region**														
Capital^b^	0.04	0.06***	0.38	0.09***	0.15	0.08***	−0.07	0.05***	−0.10	0.07***	−0.18	0.13***	−0.013	0.08***
**Area**														
Rural^c^	−0.03	0.03*	0.04	0.05*	−0.03	0.07	0.01	0.03	−0.02	0.04	−0.03	0.09	−0.07	0.05***
**Education**														
Higher^d^	0.07	0.03***	0.10	0.05***	0.18	0.08***	0.05	0.04***	0.06	0.04***	0.07	0.04***	0.16	0.04***
**Household wealth**														
Medium^e^	0.08	0.03***	−0.04	0.07	0.10	0.06***	0.06	0.03***	−0.01	0.05	0.02	005	−0.01	0.05
Rich^e^	0.12	0.04***	−0.01	0.07	0.15	0.08***	0.08	0.04***	0.01	0.05	−0.05	0.10	0.08	0.07***
N	14,310		6,493		4,676		13,376		5,884		6,066		7,727	
Adjusted R^2^	0.12		0.19		0.15		0.09		0.02		0.13		0.07	

Notes: B = unstandardized regression coefficients: SE = Standard errors.

Estimates are weighted with sample weights.

a Not married;

b −1 region (remaining regions are controlled);

c Urban;

d Else;

e Poor.

* p ≤ 0.05,

** p ≤ 0.01,

*** p ≤ 0.001.
